# Supplementary material for: Reactogenicity to the mRNA-1273 Booster According to Previous mRNA COVID-19 Vaccination
Source: Vaccines (Basel). 2022 Jul 29;10(8):1217. doi: 10.3390/vaccines10081217 (PMC9414494; doi:10.3390/vaccines10081217)
Supplement: Supplementary file 1 [file vaccines-10-01217-s001.zip › vaccines-1832434-supplementary.pdf]

Supplementary material

Annex 1. Questionnaire

- Date of birth
- Sex: Women, Men, Non-binary
- Worker category: medical doctor, registered nurse, other (with patient contact), other (without patient contact).
- Have you ever experienced a severe allergy (anaphylactic shock or glottis edema)?: yes, no
- What caused this reaction?: medications, other
- Do you have or have had a chronic condition (such as cardiac insufficiency, ischemic heart disease, asthma, diabetes, chronic bronchitis, neurological disease, renal failure or chronic liver disease)?: yes, no
- Have you ever had COVID-19?: yes, no
- Date of COVID-19 diagnosis
- How serious were your COVID-19 symptoms?: asymptomatic infection, mild or moderate symptoms, hospitalization

- Date of vaccination dose 1
- Which COVID-19 vaccine did you receive: Pfizer, Moderna, Astra-Zeneca, Janssen, I don't know

- Date of vaccination dose 2
- Which COVID-19 vaccine did you receive: Pfizer, Moderna, Astra-Zeneca, Janssen, I don't know

- Did you have adverse reactions to COVID-19 vaccine first or secong dose: yes, no
- Which adverse reactions did you suffer (multiple choice):
    - Pain at injection site
    - Swelling or redness at injection site
    - Fatigue
    - Headache
    - Muscle or join pain
    - Chills
    - Fever (>=37.5º)
    - Nausea or vomiting
    - Adenopathy / swollen lymph nodes
    - Insomnia
    - Malaise
    - Hives or rash
- How long did the adverse reactions last (in days)?
- Did you need a medical leave because of the adverse reactions?: yes, no
- Did you need medical attention because of the adverse reactions?: yes, no
- Was the adverse reaction life-threatening?: yes, no

- Date of vaccination booster
- Which COVID-19 vaccine did you receive: Pfizer, Moderna, I don’t know
- Which adverse reactions did you suffer (multiple choice):
    - Pain at injection site
    - Swelling or redness at injection site
    - Fatigue
    - Headache
    - Muscle or join pain
    - Chills
    - Fever (>=37.5º)
    - Nausea or vomiting
    - Adenopathy / swollen lymph nodes
    - Insomnia
    - Malaise
    - Hives or rash
- How long did the adverse reactions last (in days)?
- Did you need a medical leave because of the adverse reactions?: yes, no
- Did you need medical attention because of the adverse reactions?: yes, no
- Was the adverse reaction life-threatening?: yes, no

- Do you allow us to access your clinical history?: yes, no
    - What is your health record ID code?

Annex 2. Self-reported adverse reaction to COVID-19 booster comparing history of COVID-19 infection versus no history of COVID-19 infection.

Characteristic	History of COVID-19 n = 276 (%)	History of COVID-19 95% CI*	No history of COVID-19 n = 946 (%)	No history of COVID-19 95% CI*	p-value
**Some adverse reaction to booster**	245 (88.8%)	85.0%, 92.5%	836 (88.4%)	86.3%, 90.4%	0,900
**Duration of the reaction (days, median, IQR**)**	3	(2, 3)	3	(2, 4)	0,110
Pain at injection site	198 (71.7%)	66.4%, 77.1%	706 (74.6%)	71.9%, 77.4%	0,300
Swelling or redness	72 (26.1%)	20.9%, 31.3%	217 (22.9%)	20.3%, 25.6%	0,300
Fatigue	148 (53.6%)	47.7%, 59.5%	513 (54.2%)	51.1%, 57.4%	0,900
Headache	134 (48.6%)	42.7%, 54.4%	459 (48.5%)	45.3%, 51.7%	>0.999
Muscle or joint pain	116 (42.0%)	36.2%, 47.9%	360 (38.1%)	35.0%, 41.1%	0,200
Chills	142 (51.4%)	45.6%, 57.3%	451 (47.7%)	44.5%, 50.9%	0,300
Fever (≥37.5º)	123 (44.6%)	38.7%, 50.4%	367 (38.8%)	35.7%, 41.9%	0,085
Nausea or vomiting	51 (18.5%)	13.9%, 23.1%	148 (15.6%)	13.3%, 18.0%	0,300
Adenopathy	35 (12.7%)	8.76%, 16.6%	142 (15.0%)	12.7%, 17.3%	0,300
Insomnia	26 (9.4%)	5.97%, 12.9%	96 (10.1%)	8.22%, 12.1%	0,700
Malaise	170 (61.6%)	55.9%, 67.3%	546 (57.7%)	54.6%, 60.9%	0,200
Hives or rash	1 (0.4%)	0%, 1.07%	17 (1.8%)	0.95%, 2.64%	0,093
**Medical leave after booster**	54 (22.0%)	16.9%, 27.2%	159 (19.0%)	16.4%, 21.7%	0,300
**Potential life threatening reaction to booster**	0 (0.0%)		0 (0.0%)		
**Perception of booster more reactogenic**	144 (58.8%)	52.6%, 64.9%	486 (58.1%)	54.8%, 61.5%	0,900

*95% CI: 95% confidence interval. ** IQR: interquartile range.
